# Finnish Translation and Linguistic Validation of the CLEFT-Q Questionnaire

**DOI:** 10.1177/10556656231162454

**Published:** 2023-03-07

**Authors:** Mikael Westerlund, Arja Heliövaara, Junnu Leikola, Pauliina Homsy

**Affiliations:** 1 Department of Plastic Surgery, 3835 University of Helsinki and 159841 Helsinki University Central Hospital, Helsinki, Finland; 2 Department of Plastic Surgery, Cleft Palate and Craniofacial Center, 3835 University of Helsinki and 159841 Helsinki University Central Hospital, Helsinki, Finland

**Keywords:** quality of life, esthetics, psychosocial adjustment, scarring

## Abstract

**Objective:**

Cleft lip and/or palate (CL/P) can have long-lasting effects on the appearance, function, and psychosocial wellbeing of patients. The CLEFT-Q questionnaire is a patient-reported outcomes instrument specifically designed to assess the health-related quality of life of patients with CL/P. The aim of this study was to produce and linguistically validate a Finnish version of the CLEFT-Q questionnaire.

**Design:**

The CLEFT-Q questionnaire was translated into Finnish following guidelines of the International Society for Pharmacoeconomics and Outcomes Research. Pilot testing with cognitive debriefing interviews was conducted on patients of the target age range of the questionnaire, 8–29, and with various cleft types.

**Results:**

The CLEFT-Q questionnaire translated readily into Finnish. A review of the backward translation led to two words being changed. Thirteen patients – ten females and three males – with a median age of 14 years, participated in the cognitive debriefing interviews. The interviews led to further nine word changes. The pilot study data suggested that the performance of the Finnish version of the instrument is in line with the original CLEFT-Q questionnaire.

**Conclusions:**

The Finnish version of CLEFT-Q produced here is linguistically valid and ready for use in the evaluation of the health-related quality of life of patients with CL/P. However, future work is needed to further assess the validity and the reliability of the CLEFT-Q in the Finnish patient population.

## Introduction

Cleft lip and/or palate (CL/P) is one of the most common developmental disorders, with over 10 million people affected worldwide and an incidence of 7.9–14.2 per 10 000.^1–3^ Finland has a slightly higher incidence of CL/P, 20 per 10 000.^4^ Depending on the type and extent of the cleft, CL/P and its treatments can affect eating and speaking, facial appearance, self-esteem, and psychosocial wellbeing.^5,6^ The goal of cleft treatment is to normalize a patient's function and appearance.^7,8^ However, patients can be left with a visible scar, asymmetry, maxillary hypoplasia and crossbite, or problems with the function of the soft palate. Thus, CL/P can affect patients’ health-related quality of life.^9–12^

Several patient-reported outcome measures, such as the Satisfaction With Life Scale and Psychosocial Impact of Dental Aesthetics Questionnaire, have been used for patients with CL/P in clinical practice.^13–17^ Mainly designed for other patient groups, these instruments are not specific enough to capture all aspects of health-related quality of life in patients with CL/P.^13,18^ In Finland, for example, a non-validated questionnaire with 15 items is currently used for measuring the impact of the cleft and cleft treatments in patients with CL/P. While including questions on topics relevant to cleft patients, such as facial appearance, it does not address any functional or social aspects. Further, the range of questionnaires used in the assessment of patients with CL/P has limited the ability to compare treatment outcomes across centers and countries.

The CLEFT-Q questionnaire is a patient-reported outcomes instrument developed specifically for patients with CL/P.^19^ It covers aspects such as appearance, facial function, speech, and social distress.^9^ The questionnaire consists of twelve scales and one checklist. The questionnaire has been psychometrically validated with 2434 patients from 12 countries and has since been translated into several languages.^20–22^ It has also been shown to be psychometrically valid when used on children and young adults with a range of other facial conditions.^23^ The aim of this study was to produce and linguistically validate a Finnish version of the CLEFT-Q questionnaire.

## Methods

The CLEFT-Q questionnaire was translated into Finnish following the instructions of the International Society for Pharmacoeconomics and Outcomes Research (ISPOR) and according to the Mapi Research Trust guidelines.^24,25^ Permission for the translation was sought from the license owners.^26^ The study was approved by the ethics committee of Helsinki University Hospital, Finland (HUS/917/2021).

**Figure 1.**
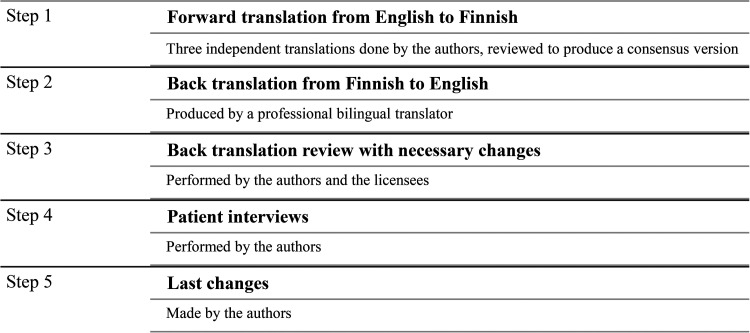
Steps of the translation process of the CLEFT-Q questionnaire.

The translation process consisted of five steps (Figure 1). The original English CLEFT-Q questionnaire was first translated into Finnish by three of the authors (PH, AH, JL). All the translating authors are native Finnish speakers with fluent English, and they are familiar with the subject area. The Finnish version was translated back into English by a professional bilingual translator. This backward translation was then reviewed by the authors and the developers of CLEFT-Q. Any ambiguities were identified and resolved.

A pilot study was conducted to assess the linguistic validity of the newly translated Finnish CLEFT-Q questionnaire. Patients across the target age range of the questionnaire, 8–29 years old, were recruited during 2021 from the outpatient clinic of the Cleft Palate and Craniofacial Center, Department of Plastic Surgery, Helsinki University Hospital, Finland. All participants received written information about the study, and the participants, as well as guardians of children under 15 years old, provided written, informed consent for the study. For children, their guardian was present in the interview room during most of the interviews, based on the patients’ request.

The participants filled out sections of the CLEFT-Q questionnaire relevant to their medical history. For example, patients without a lip scar did not fill out the lip scar module. A Cognitive Debriefing Interview Guide, provided by CLEFT-Q developers, was used for the interviewing. The questions and answer options were then discussed with the patient, with the aim of assessing the understandability of the questionnaire. Any difficulties in language comprehension were recorded. The patients were also invited to suggest alternative expressions for any challenging words and concepts they had identified. Patient records were reviewed for participants’ age, sex, and cleft type.

The data from the 12 scales in the pilot study were converted to a scale from 0 (worst) to 100 (best) using the Rasch conversion scale from the original publication.^9^ The Eating and drinking-checklist does not fit the Rasch model but functions as a checklist and, therefore, a direct score from 9 (worst) to 36 (best) was used.^9,27^ All data were analyzed with SPSS.^28^ The data are presented as median, maximum, and minimum values in Tables 3 and 4.

## Results

### Linguistic Translation

The translation process began with forward translation. Comparison of the three forward translations identified six words with frequently used synonyms that had been translated inconsistently; for these, the word assessed as the easiest to understand for children was chosen for use. For example, there are two possibilities for the word “biting” in Finnish – “haukata” or “puraista”. The word “haukata” was chosen because it is considered more commonly used among children. The adjective “symmetrical”, or “symmetrinen” in Finnish, was judged too uncommon for children to reliably understand. Being a direct and accurate translation, the word was left in parentheses, while a simpler word, “samanlainen”, was used in the main sentence.

The review of the backward translation led to two changes. In the School function scale item 1, a personal pronoun “I” was added. In the Eating and drinking checklist item 2, “suck through a straw” was changed to “drink through a straw”.

### Cognitive Debriefing Interviews

Thirteen native Finnish speakers, – ten females and three males – with a median age of 14 years, and with different cleft types participated in the cognitive debriefing interviews (Table 1). One of the participants, a ten-year-old girl with no pertinent medical history, did not notice the sentence “How much do you like…” at the beginning of some of the scales, and their answers were subsequently excluded. Another participant, a 15-years old girl, was unable to attend the interview due to a scheduling issue, but their answers were included in the pilot results. Thus, thirteen patients were included in both the interviews and the pilot data.

**Table 1.** Details of the Pilot Study Patients.

Patient ID	Age (years)	Sex (M/F)	Cleft type
1	14	F	UCLP
2	12	F	CP
3	17	F	UCL
4	14	F	BCL + SMCP
5	12	M	CP
6	8	M	UCLP + UCL
7	19	F	UCLP
8	10	F	CP
9	13	F	UCLP
10	24	F	CP
11	29	F	UCLP
12	17	F	SMCP
13	15	F	CP
14	14	M	UCLP

Age (mean 16 years, median 14 years, mode 14 years). Patient ID = 13 did not participate in the interview. M, male; F, female; CP, cleft palate; SMCP, submucous cleft palate; UCL, unilateral cleft lip; BCL, bilateral cleft lip; UCLP, unilateral cleft lip and palate.

Nine modifications were made to the Finnish version of CLEFT-Q based on the cognitive debriefing interviews (Table 2). The aim of the corrections was to make the language simpler and more understandable for children. For example, the words “party”, “oval” and “jaws” were commented on by the children as being too difficult. In addition, the instruction “Answer each question by circling one number.” was missing in the pilot questionnaire and was added to every scale in the final version. In the final version of the questionnaire, key words were also underlined.

**Table 2.** Items Modified After the Cognitive Debriefing Interviews. Nine Changes Were Made Following the Patient Interviews. Seven of the Changes are Presented in the Table. the Remaining two Were the Underscoring of key Words and the Addition of the Instruction “Answer Each Question by Circling one Number”.

Item/scale	The original English version	The Finnish version used in the patient interviews	Comments by the participants	The final Finnish version
Face, item 2	…how your face looks when you are ready to go out (like to a party)?	…how your face looks when you go out (eg, to party)?	Younger participants had difficulties with the word “party”.	…how your face looks when you go out (eg, to party with your friends)?
Face, item 3	…the shape of your face (eg, round or oval)?	…the shape of your face (eg, round or oval)?	Younger participants had difficulties with the word “oval”.	…the shape of your face (eg, round or oval/Finnish synonym for “oval”)?
Lips, item 6	…how your lips look when your mouth is closed?	…how your lips look when your lips are closed?	It is clearer to use the word “mouth” instead of “lips” where it had been used in the original English version.	…how your lips look when your mouth is closed?
Jaws scale	Applies to the entire scale.	The word “jaws” was used.	In Finnish it is unclear if the noun “jaws” is plural or singular.	“Upper and lower jaw”.
Social function, item 6	I feel confident when I go out (like to a party).	I feel confident when I go out (eg, to party).	Younger participants had difficulties with the word “party”.	I feel confident when I go out (eg, to party with my friends).
Social function, item 8	It's easy for me to make friends.	It is easy for me to make new friends.	The word “new” was removed to keep the question similar to the original version.	It is easy for me to make friends.
Speech distress, item 1	I avoid going out because of my speech (like to a party).	I avoid going out because of my speech (eg, to a party).	Younger participants had difficulties with the word “party”.	I avoid going out because of my speech (eg, to a party with my friends).

Pilot study data and normative data from the original validation study for the CLEFT-Q scales assessing appearance are presented in Table 3, and the equivalent data for functional scales are in Table 4.^9^ The median scores of the appearance scales were between 53 and 64 out of 100, and the medians of the functional scales between 61 and 83 out of 100. For the Eating and drinking checklist, the median score was 35 out of 36, with only two patients (18%) reporting any issues.

**Table 3.** Appearance-Related Scales of CLEFT-Q. Pilot Study Data for 13 Patients are Shown in the Table as Median and Range Values. the Mean and Standard Deviation (SD) from the Original CLEFT-Q Validation study^9^ are Shown in the Right Column.

	Median	Range	n	Mean (SD) Original CLEFT-Q validation study^9^
Appearance of the face	56	42–76	13	63 (19.6)
Appearance of the lips	56	40–86	13	63 (24.9)
Appearance of the nose	55	36–100	13	59 (23.1)
Appearance of the nostrils	64	9–100	13	56 (29.2)
Appearance of the jaws	62	29–100	13	68 (26.8)
Appearance of the teeth	58	34–73	13	55 (23.7)
Appearance of the cleft lip scar	53	30­­–79	8	58 (28.3)

**Table 4.** Function-Related Scales of CLEFT-Q. Pilot Study Data for 14 Patients are Shown in the Table as Median and Range Values. The Mean and Standard Deviation (SD) from the Original CLEFT-Q Validation study^9^ are Shown in the Right Column.

	Median	Range	n	Mean (SD) Original CLEFT-Q validation study^9^
Psychological function	61	54–86	14	74 (18.9)
School function	75	58–91	12	76 (17.7)
Social function	71	58–84	13	73 (17.3)
Speech distress	83	49–100	13	69 (21.3)
Speech function	80	66–100	13	69 (21.3)

## Discussion

A cleft lip and/or palate can affect a patient's health-related quality of life, self-esteem, and function.^5,9,29^ Here we produced a Finnish version of the patient-reported outcome instrument CLEFT-Q and demonstrated its linguistic validity in a pilot study with 13 patients. CLEFT-Q covers essential aspects of the health-related quality of life of patients with CL/P.^9^ Having been translated into several languages, CLEFT-Q enables comparison between patients from different countries with various native languages.^20,21^ This represents a significant development in facilitating international research collaboration on this relatively small patient group.

Only a few word changes were required during the translation process of CLEFT-Q into Finnish. The pilot study included patients from the recommended age range, 8–29 years old. Many of the patients spontaneously gave positive feedback on the questionnaire items and acknowledged the potential of the new questionnaire. In particular, patients were pleased that both psychological and appearance factors were addressed in the questions. However, a few parents commented that the questionnaire was excessively focused on facial appearance.

Normative CLEFT-Q scale values for patients of different ages and cleft types have been provided by the CLEFT-Q questionnaire developers.^9,30^ Results of the appearance scales for our pilot population were in line with the mean values reported in the large international study during which the questionnaire was developed.^9^ Scores for the three scales related to function differed between our study and the original validation study.^9^ The median psychological function score was lower, and the speech distress and the speech function scores were higher in our population than reported in the original validation study. The difference in speech distress and speech function is likely to have arisen from the validation study having included only patients with speech problems, while the scales were answered by all of our pilot patients, regardless of their speech performance. However, our pilot population of 13 patients was too small for formal statistical analysis or any subgroup evaluation. In addition, minimal clinically important difference values have not yet been established for the CLEFT-Q scales, further limiting interpretation of the differences observed in the scores.

The main limitations of our study are its qualitative nature and the small study group. In addition, our study population did not include patients with bilateral clefts. The youngest participants struggled to focus toward the end of the CLEFT-Q questionnaire and the subsequent interview. Therefore, the scales may not have been accurately completed by these patients. In addition, one of the patients failed to compete the second half of the questionnaire due to getting too distracted. It is also possible that the presence of guardians may have affected childreńs answers, including their willingness to comment on words they found difficult to understand. In the original validation study the answers from participants with guardians present were excluded.^30^ Even though both sexes were represented in the pilot study, only a fifth of the participants were men. In addition, patients with cleft palate were underrepresented in the pilot population (36%); this is actually the most common cleft type in Finland, affecting 60% of patients with CL/P.^31^ The pilot study did not include patients with bilateral cleft lip and palate, a group that historically scores lower on CLEFT-Q subscales.^30^ With linguistic validation being only the first step in adopting an outcomes instrument in a new language and cultural environment, future studies are needed to establish the psychometric reliability and validity of the Finnish CLEFT-Q.

## Conclusion

In conclusion, the newly produced, culturally adapted, and linguistically validated Finnish version of CLEFT-Q is easily understandable by patients with cleft lip and/or palate across the questionnaire target age range. The pilot population results were in line with values reported for postoperative patients in the original, international cohort. The Finnish version of CLEFT-Q is available free of charge at the Q-Portfolio website for use in future research and clinical practice.^26^
